# Artificial intelligence-estimated biological heart age using a 12-lead electrocardiogram predicts mortality and cardiovascular outcomes

**DOI:** 10.3389/fcvm.2023.1137892

**Published:** 2023-04-13

**Authors:** Yong-Soo Baek, Dong-Ho Lee, Yoonsu Jo, Sang-Chul Lee, Wonik Choi, Dae-Hyeok Kim

**Affiliations:** ^1^ Division of Cardiology, Department of Internal Medicine, Inha University College of Medicine and Inha University Hospital, Incheon, South Korea; ^2^ DeepCardio Inc., Incheon, South Korea; ^3^ School of Computer Science, University of Birmingham, Birmingham, United Kingdom; ^4^ Department of Computer Engineering, Inha University, Incheon, South Korea; ^5^ Department of Information and Communication Engineering, Inha University, Incheon, South Korea

**Keywords:** ECG age, heart age, biological ageing, artificial intelligence, mortality, hospitalization, MACE

## Abstract

**Background:**

There is a paucity of data on artificial intelligence-estimated biological electrocardiography (ECG) heart age (AI ECG-heart age) for predicting cardiovascular outcomes, distinct from the chronological age (CA). We developed a deep learning-based algorithm to estimate the AI ECG-heart age using standard 12-lead ECGs and evaluated whether it predicted mortality and cardiovascular outcomes.

**Methods:**

We trained and validated a deep neural network using the raw ECG digital data from 425,051 12-lead ECGs acquired between January 2006 and December 2021. The network performed a holdout test using a separate set of 97,058 ECGs. The deep neural network was trained to estimate the AI ECG-heart age [mean absolute error, 5.8 ± 3.9 years; R-squared, 0.7 (*r* = 0.84, *p* < 0.05)].

**Findings:**

In the Cox proportional hazards models, after adjusting for relevant comorbidity factors, the patients with an AI ECG-heart age of 6 years older than the CA had higher all-cause mortality (hazard ratio (HR) 1.60 [1.42–1.79]) and more major adverse cardiovascular events (MACEs) [HR: 1.91 (1.66–2.21)], whereas those under 6 years had an inverse relationship (HR: 0.82 [0.75–0.91] for all-cause mortality; HR: 0.78 [0.68–0.89] for MACEs). Additionally, the analysis of ECG features showed notable alterations in the PR interval, QRS duration, QT interval and corrected QT Interval (QTc) as the AI ECG-heart age increased.

**Conclusion:**

Biological heart age estimated by AI had a significant impact on mortality and MACEs, suggesting that the AI ECG-heart age facilitates primary prevention and health care for cardiovascular outcomes.

## 1. Introduction

Increasing age is the strongest determinant of mortality and cardiovascular disease (CVD) (1). Chronological age (CA) is the number of years that have passed since birth. In contrast, biological age, caused by a gradual accumulation of impairments to the body's cells and tissues, also known as the physiological and functional age, is distinct from CA (2). However, biological aging may be evaluated over a longer time than CA if biological processes, tissues, and organs age at a faster-than-average rate (3). CVD frequently proceeds asymptomatically for many years, contributing significantly to mortality, cardiovascular clinical outcomes, and related pathological processes (4). Therefore, biological age should be included in CVD risk assessments, particularly for primary prevention. Although several biomarkers of biological age, including the biochemical, molecular, and genetic biomarkers, have been identified, it remains unclear which best measures aging (5). It might be challenging for healthcare service users to interpret the hazards of multiple biochemical, molecular, and genetic biomarkers regarding primary prevention. As the paradigm shifts from treatment-centered to prevention-centered medicine, a demand exists for an alternative strategy that healthcare users can readily understand and apply to prevent CVD (6).

Twelve-lead electrocardiography (ECG) is a screening tool for heart disease. Although ECG interpretation requires expert knowledge and experience, artificial intelligence (AI)-enhanced ECG using deep neural networks may discover incomprehensible signals and patterns to humans, making it a robust, non-invasive biomarker. In addition, the AI-based ECG may predict mortality, arrhythmias, heart failure, valvular heart disease, and electrolyte abnormalities (7–13). Recent studies (8, 13, 14) regarding AI-based ECG have reported that biological age is distinct from the CA. However, data on the AI-estimated biological ECG heart age (AI ECG-heart age) for predicting cardiovascular outcomes are still lacking. We previously presented (10) a new deep-learning network employing digitized ECG raw data. In this study, we investigated whether AI ECG-heart age prediction using large-scale 12-lead ECG raw data could predict cardiovascular-related hospitalization and cardiovascular rates.

## 2. Materials and methods

### 2.1. Study design and patient selection

**Figure 1:**
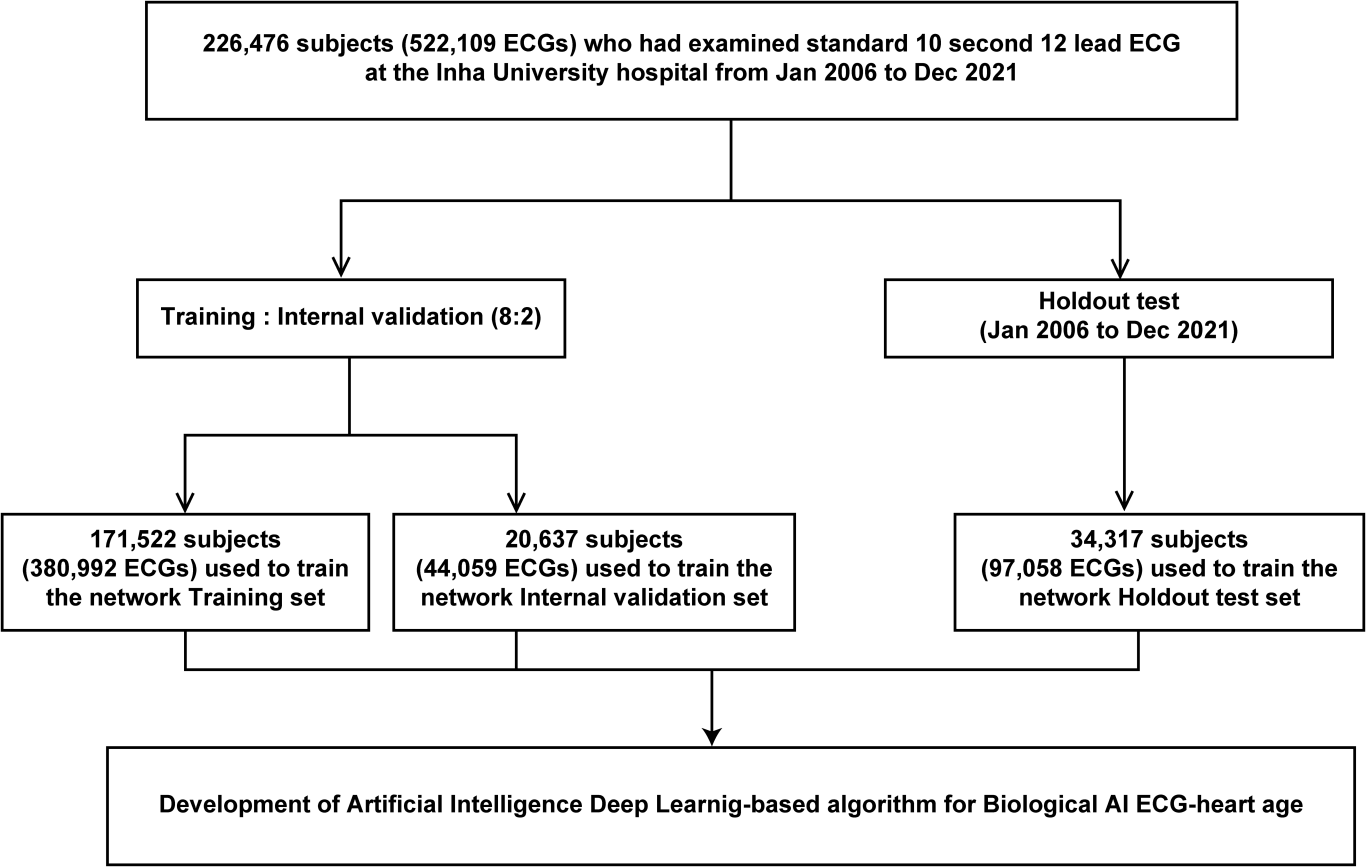
Creation of the study datasets. ECG, electrocardiography.

We used 12-lead ECG data, mostly from patients at the Health Examination Center, Inha University Hospital. Overall, 226,476 adults (age ≥18 years) who underwent standard 12-lead ECG (men, 118,559; 52.3%, mean age 47.2 ± 20.6 years) from January 2006 to December 2021 were retrospectively included. Figure 1 shows the use of training, validation, and test set cases within this population. We extracted the XML raw data of all ECGs and used it for deep learning. Furthermore, all ECGs were collected using a GE-Marquette ECG machine (Marquette Tools, Milwaukee, WI, USA). Raw data were stored as XML documents using the MUSE data management system for databases. This study was non-invasive and did not require patient consent; the study protocol was approved by the Institutional Review Board of Inha University Hospital (2021-10-006) and adhered to the principles of the Declaration of Helsinki. In addition, the ethical committee of Inha University Hospital approved all analyses, and the STARD guidelines were employed.

### 2.2. Development of an AI-based ECG heart age model

**Figure 2:**
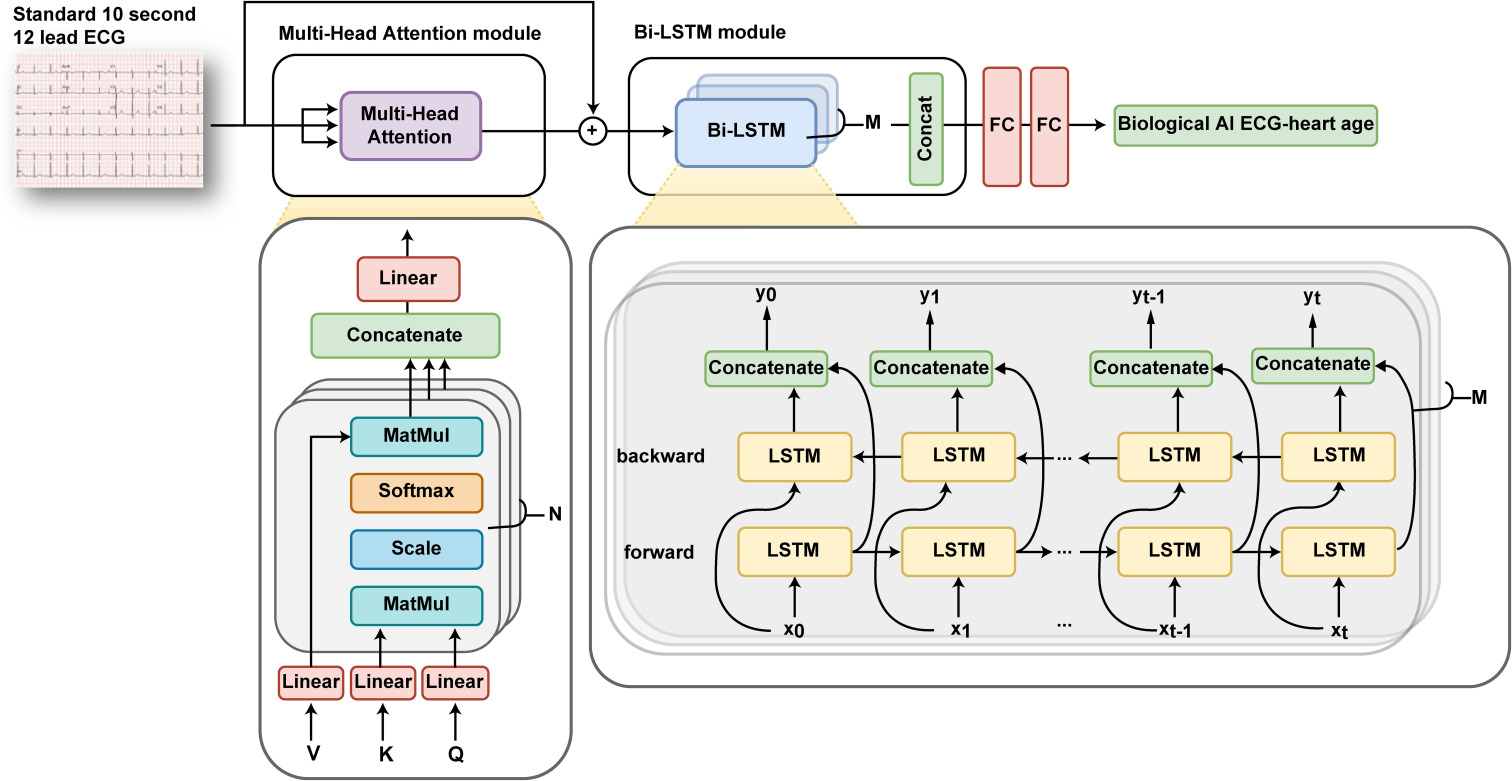
Overview of an artificial intelligence-based age prediction algorithm using a 12-lead electrocardiogram. ECG, electrocardiography.

The model receives eight leads (I, II, V1, V2, V3, V4, V5, and V6) of ECG data (sampling rate, 500 samples/s) taken for 10 s as input and output AI-estimated ECG heart age. Notably, the values from the remaining four leads (III, aVR, aVL, and aVF) were not included as inputs, as they can be mathematically derived using the values of leads I and II. The initial input values were weighed to important features through Multi-head attention and learned through bi-directional long short-term memory model blocks (Figure 2). The model loss function is the mean squared error, and the optimizer is the Adam (learning rate = 0.001, *β* = 0.9).

### 2.3. Clinical outcomes analysis

We applied the AI ECG-heart age algorithm to the test set and investigated its relationship with clinical outcomes, including all-cause death, cardiovascular-related mortality, and major adverse cardiovascular events (MACE). Based on 5.8 ± 3..9 years of the mean absolute error (MAE) in our data, we classified patients into three groups based on their AI-estimated ECG age: those who were ≥6 years older than the CA, those who were within 6 years of the CA, and those who were ≤6 years younger than the CA.

### 2.4. Statistical analysis

Continuous and categorical variables are reported as mean ± standard deviation and percentage and frequency, respectively. Categorical variables were compared using chi-square tests, and continuous variables were compared using one-way analysis of variance (ANOVA) for each of the groups. Univariate correlations between variables were assessed by Pearson's correlation coefficients (r). Linear regression analyses were performed to assess the relationships between AI-ECG heart age and ECG findings including PR interval, QRS duration, QT interval and QTc. In addition, we performed Cox proportional-hazard regression analysis to assess the clinical impact of the difference between biological heart age as predicted by AI and CA. For all variables, *p* < 0.05 was considered statistically significant. All statistical analyses were performed using the statistical package for the social sciences (version 26.0; IBM Corp., Armonk, NY, USA) and Python.

## 3. Results

### 3.1. Baseline characteristics of the study population

Table 1 summarizes the baseline characteristics, comorbidities of the training set, development of the internal validation datasets and holdout test sets, and ECG findings. The mean age of the study population was 47.2 ± 20.6 years, and 52.3% were men. The mean body mass index was 24.3 ± 13.7 kg/m^2^. The proportions of hypertension (HTN), diabetes mellitus (DM), heart failure (HF), and stroke were 5.2%, 4.0%, 1.1%, and 3.8%, respectively. The heart rate per minute was 80.5 ± 41.4, and the PR interval, QRS duration, and corrected QT Interval (QTc) were 159.8 ± 28.3, 92.6 ± 16.3, and 432.6 ± 34.4, respectively.

**Table 1:** Characteristics of the patients and electrocardiographic findings in the training, validation, and test sets.

Characteristics	Training set (*n* = 171,522)	Validation set (*n* = 20,637)	Test set (*n* = 34,317)	*p*-value
Chronological age, years	47.11 ± 20.67	46.12 ± 20.54	48.13 ± 20.79	<0.05
Sex, male, *n* (%)	87,467 (50.9)	10,457 (50.6)	20,635 (51.3)	0.2
Height, cm	163.06 ± 10.64	163.39 ± 9.73	162.97 ± 9.83	0.55
Weight, kg	64.02 ± 14.33	64.56 ± 13.72	63.83 ± 16.64	0.36
BMI, (kg/m^2^)	24.36 ± 14.73	24.37 ± 13.0	24.02 ± 9.29	0.52
Obesity (BMI ≥ 25 kg/m^2^), %	3,660 (2.29)	462 (2.4)	907 (2.41)	0.46
DM, *n* (%)	6,301 (3.94)	768 (4.01)	1,623 (4.23)	<0.05
HTN, *n* (%)	8,241 (5.16)	1,005 (5.26)	2,041 (5.43)	0.07
Dyslipidemia	4,691 (2.93)	535 (2.80)	1,103 (2.93)	0.54
HF, *n* (%)	1,788 (1.12)	225 (1.17)	512 (1.36)	<0.05
Stroke (ischemic, hemorrhagic)/TIA), *n* (%)	6,089 (3.81)	717 (3.75)	1,536 (4.09)	<0.05
MI, *n* (%)	1,458 (0.91)	184 (0.96)	363 (0.96)	0.48
Vascular disease (PAOD), *n* (%)	1,218 (0.76)	139 (0.72)	300 (0.79)	0.55
CKD, *n* (%)	2,263 (1.41)	231 (1.20)	613 (1.63)	<0.05
**ECG findings**				
Heart rate, bpm	80.22 ± 30.88	79.59 ± 41.01	82.15 ± 43.67	<0.05
PR interval, ms	159.98 ± 28.21	159.68 ± 28.80	159.61 ± 28.34	<0.05
QRS duration, ms	92.57 ± 16.07	92.67 ± 15.97	92.83 ± 17.36	<0.05
QT interval, ms	393.76 ± 41.68	394.13 ± 40.53	393.30 ± 43.49	<0.05
QTc, ms	432.36 ± 34.07	430.72 ± 33.11	434.79 ± 36.32	<0.05
*P* wave axis	48.68 ± 23.6	48.58 ± 23.47	48.47 ± 24.16	<0.05
R wave axis	40.30 ± 39.38	41.29 ± 38.46	39.72 ± 40.38	<0.05
T wave axis	46.08 ± 40.07	45.13 ± 37.66	47.26 ± 42.60	<0.05

Values are expressed as *n* (%) or means ± standard deviations. *p*-value of ANOVA or *χ*^2^ test among the training, validation, and test datasets. BMI, body mass index; bpm, beats per minute; CKD, chronic kidney disease; DM, diabetes mellitus; ECG, electrocardiography; HF, heart failure; HTN, hypertension; MI, myocardial infarction; PAOD, peripheral arterial occlusive disease; QTc, the corrected QT Interval; TIA, transient ischemic attack.

### 3.2. Relationship between CA and the AI-estimated biological ECG heart age

**Figure 3:**
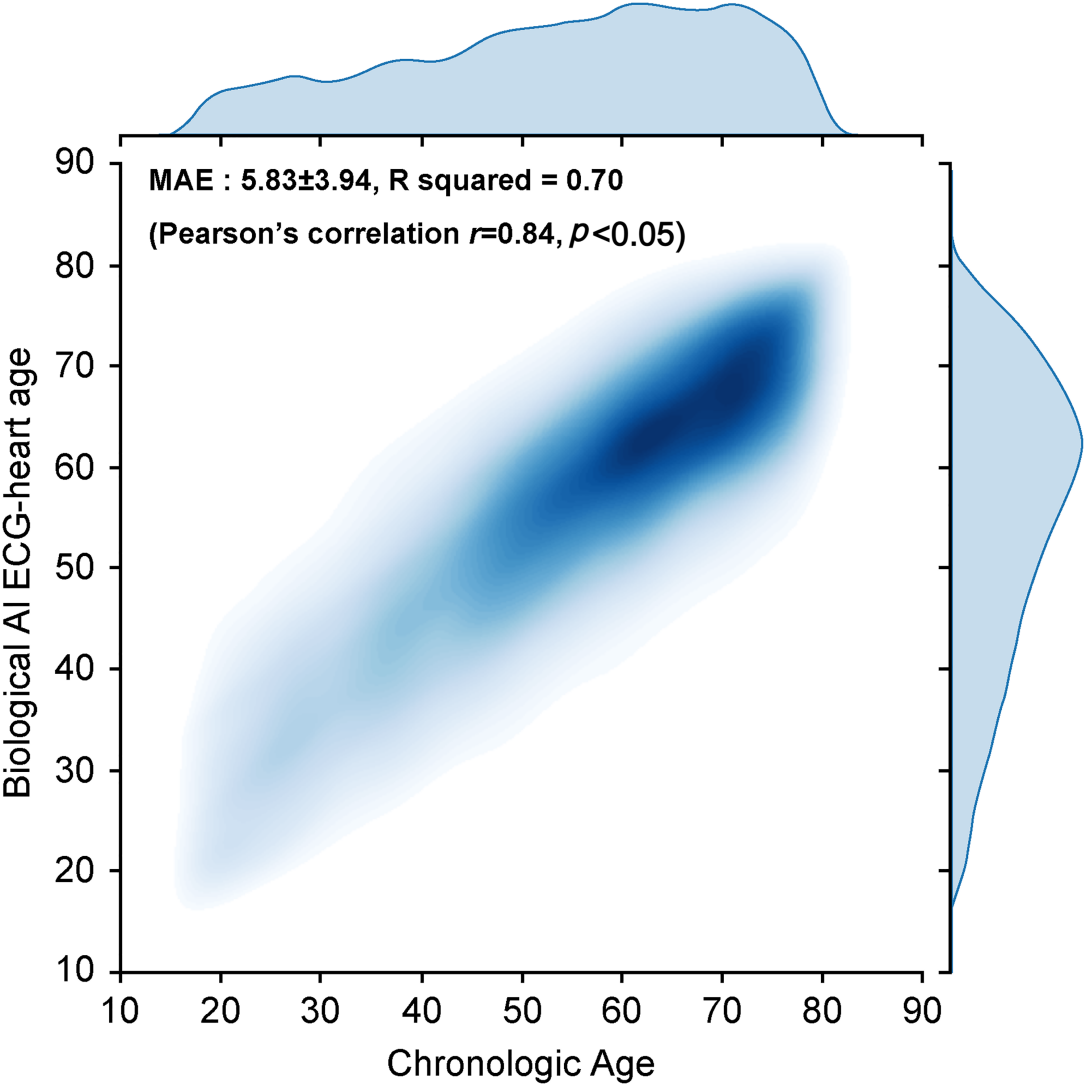
Correlation between the AI-estimated biological ECG heart age and chronological age shown *via* a heat map. AI, artificial intelligence; ECG, electrocardiography; MAE, mean absolute error.

We developed an AI algorithm using raw ECG data set comprising 522,109 ECGs (226,476 patients) to estimate the biological heart age. The training and internal validation sets were allocated at an 8:2 ratio [380,992 (171,522 patients) and 20,637 (44,059 patients) ECGs for the training set and internal validation set, respectively]. In addition, we performed external validation using a holdout test set [97,058 ECGs (34,317 patients)] from January 2006 to December 2021 (Figure 1). For the holdout data set, MAE of the difference between the CA and AI-estimated biological ECG age was 5.8 ± 3.9 years with an *R*-squared of 0.7 (*r* = 0.84, *p* < 0.05). Figure 3 shows a heat map of the CA vs. AI-estimated biological ECG age.

### 3.3. AI-estimated biological ECG age and prediction of cardiovascular outcomes

**Figure 4:**
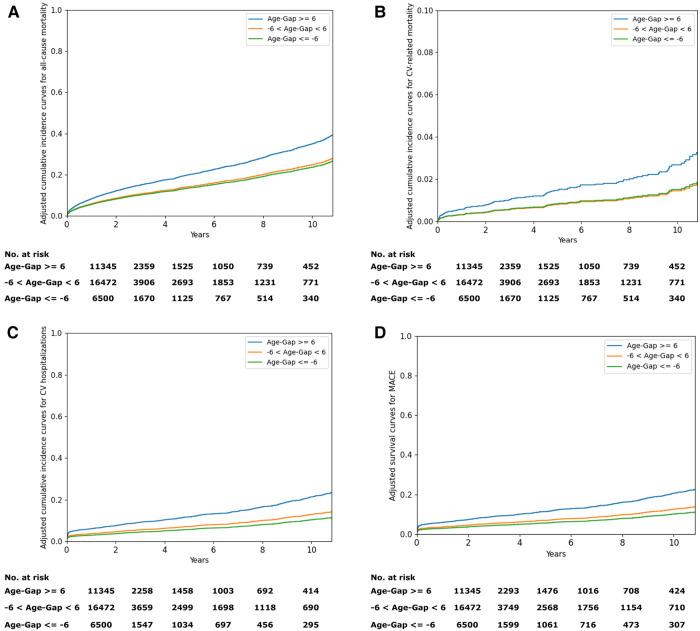
(**A**) Adjusted cumulative incidence curves for all-cause mortality. (**B**) Adjusted cumulative incidence curves for cardiovascular-related mortality. (**C**) Adjusted cumulative incidence curves for cardiovascular hospitalizations. (**D**) Adjusted survival curves for MACE. MACE, major adverse cardiovascular events.

Figure 4 shows the adjusted cumulative incidence curves from the age and sex-adjusted Cox proportional model for all-cause mortality and cardiovascular outcomes. The relationship between the difference between the AI-estimated ECG age and CA and the mortality and cardiovascular outcomes is graphically shown using penalized B-spline curves fitted to the Cox proportional hazards model in the overall population and sex-stratified population, respectively (Supplementary Figure S1). The spline curves according to the gap between the AI-ECG heart age and CA and the hazard ratio (HR) of the all-cause mortality and cardiovascular outcomes are presented in Supplementary Figure S1. A non-linear J-shaped association was found in the gap between the AI-ECG heart age and CA variables and the all-cause mortality and CVD. Univariate Cox regression models showed significant hazard ratios (HRs) for chronological age, gender, DM, HTN, HF, stroke, myocardial infarction (MI), and chronic kidney disease (CKD), as well as AI-ECG heart age in predicting all-cause and cardiovascular-related outcomes. Notably, a 5-year increase in AI-ECG heart age was associated with HRs ranging from 1.28 (1.24–1.31), *p* < 0.05] to 1.45 (1.31–1.62), *p* < 0.05] for all-cause mortality and cardiovascular-related mortality, respectively (Supplementary Table S1). In the Cox proportional hazards models, after adjusting for relevant comorbidity factors, the patients with an AI-biological ECG age of ≥6 years greater than the CA had higher all-cause mortality [HR: 1.60, (1.42–1.79), *p* < 0.05], cardiovascular-related mortality [HR: 2.20 (1.42–3.42), *p* < 0.05], cardiovascular hospitalizations [HR: 1.93 (1.67–2.22), *p* < 0.05], and MACE [HR: 1.91, (1.66–2.21), *p* < 0.05]. Although when the ECG age was ≤6 years younger than the CA, the risk of all-cause mortality [HR: 0.82 (0.75–0.91), *p* < 0.05], cardiovascular hospitalizations [HR: 0.77 (0.67–0.88), *p* < 0.05], and MACE [HR: 0.78 (0.68–0.89), *p* < 0.05] decreased; however, the risk of cardiovascular-related mortality was not statistically significant [HR: 0.87 (0.61–1.25), *p* = 0.46] (Table 2).

**Table 2:** Hazard ratios of clinical outcomes.

	All-cause mortality		Cardiovascular-related mortality		Cardiovascular hospitalization		MACE	
**Adjusted by age and sex**	HR (95% CI)	*p*-value	HR (95% CI)	*p*-value	HR (95% CI)	*p*-value	HR (95% CI)	*p*-value
Age gap ≥6 years	1.56 (1.39–1.75)	<0.05	2.19 (1.41–2.74)	<0.05	2 (1.74–2.31)	<0.05	1.99 (1.73–2.30)	<0.05
−6 years <Age gap <6 years	1 (reference)	NA	1 (reference)	NA	1 (reference)	NA	1 (reference)	NA
Age gap ≤−6 years	0.88 (0.80–0.97)	<0.05	0.89 (0.60–1.27)	0.53	0.71 (0.62–0.81)	<0.05	0.71 (0.63–0.82)	<0.05
**Adjusted by age, sex, DM, and HTN**	HR (95% CI)	*p*-value	HR (95% CI)	*p*-value	HR (95% CI)	*p*-value	HR (95% CI)	*p*-value
Age gap ≥6 years	1.58 (1.41–1.78)	<0.05	2.20 (1.42–3.41)	<0.05	1.97 (1.71–2.27)	<0.05	1.95 (1.69–2.25)	<0.05
−6 years <Age gap <6 years	1 (reference)	NA	1 (reference)	NA	1 (reference)	NA	1 (reference)	NA
Age gap ≤−6 years	0.86 (0.78–0.95)	<0.05	0.87 (0.61–1.25)	0.46	0.75 (0.66–0.88)	<0.05	0.76 (0.66–0.87)	<0.05
**Adjusted by age, sex, DM, HTN, HF, stroke, MI, and CKD**	HR (95% CI)	*p*-value	HR (95% CI)	*p*-value	HR (95% CI)	*p*-value	HR (95% CI)	*p*-value
Age gap ≥6 years	1.60 (1.42–1.79)	<0.05	2.20 (1.42–3.42)	<0.05	1.93 (1.67–2.22)	<0.05	1.91 (1.66–2.21)	<0.05
−6 years <Age gap <6 years	1 (reference)	NA	1 (reference)	NA	1 (reference)	NA	1 (reference)	NA
Age gap ≤−6 years	0.82 (0.75–0.91)	<0.05	0.87 (0.61–1.25)	0.46	0.77 (0.67–0.88)	<0.05	0.78 (0.68–0.89)	<0.05

The table presents the hazard ratios (HR) according to the differences between AI-ECG heart age and chronological age. The HR summarizes the Cox regression models after adjusting relevant risk factors. AI, artificial intelligence; CI, confidence interval; CKD, chronic kidney disease; DM, diabetes mellitus; ECG, electrocardiography; HF, heart failure; HTN, hypertension; HR, hazard ratio; MACE, major adverse cardiovascular events; MI, myocardial infarction; NA, not applicable.

### 3.4. Analysis of ECG features based on AI-ECG heart age

We analyzed the ECG features based on the AI-ECG heart age and observed a significant increase in the PR interval, QRS duration, QT interval, and QTc interval as the AI ECG-heart age increased (all *p* for trend <0.05, Supplementary Figure S2). Specifically, we observed a significant increase in the mean PR interval (155.38 ± 21.53 ms to 173.54 ± 36.97 ms), mean QRS duration (from 90.48 ± 11.48 ms to 97.82 ± 24.79 ms), mean QT interval (392.02 ± 32.61 ms to 413.26 ± 49.18 ms), and mean QTc (422.79 ± 25.29 ms to 451.47 ± 40.62 ms) in individuals with an AI-ECG heart age between 40 and 70 years or older (Supplementary Table S2).

## 4. Discussion

### 4.1. Main findings

We developed a deep neural network to estimate AI-ECG heart age using digital raw data from a large-scale 12-lead standard ECG. The AI algorithm indicated that the discrepancy between the AI-ECG heart age and CA was associated with all-cause mortality, cardiovascular mortality, and MACE after adjusting for relevant cardiovascular risk factors. Moreover, the analysis of ECG features demonstrated significant changes in PR interval, QRS duration, QT interval, and QTc interval as AI ECG-heart age increased.

### 4.2. Biomarkers for estimating biological age

Biological age (BA) has been suggested as an alternative to CA to assess the precise aging states of individuals with various “multiple clock” biological aging. Different biological measures have been studied to construct more comprehensive models of the aging process compared with CA (15). Regarding molecular and cellular biomarkers, BA can be estimated using telomere length, deoxyribonucleic acid methylation age, epigenetic clock, inflammatory markers, and transcriptomic-, proteomic-, and metabolomics-based biomarkers, among others in the blood, urine, or feces (16, 17). The age-related structural and functional changes include increased arterial stiffness and atherosclerosis (characterized by accumulation of lipid-rich plaque in the intima and may result in an acute myocardial infarction or stroke). In addition, the carotid-to-femoral pulse wave velocity, blood pressure, endothelial dysfunction, intimal thickening, coronary artery calcium score of the heart, and other variables may be used to quantify these aging-related alterations, which are key contributors to atherogenesis (16–22). The composite biomarker predictors were developed by combining the molecular, physiological, biochemical, structural, and functional factors, such as the Klemera–Doubal Method Biological Age and Frailty indices (23). A novel method has been recently reported for a phenotypic assessment of cardiovascular aging using cardiovascular magnetic resonance radiomics measures of ventricular shape and myocardial character (24). Although various biomarkers of biological age have been studied, the association with age-related diseases is not as robust as anticipated (23). Therefore, further studies are needed to evaluate, improve, and create more accurate aging biomarkers. Since many variables, including genetics, lifestyle, environment, and other factors, might affect the risk of cardiovascular morbidity and mortality, recommendations for primary cardiovascular prevention based on risk assessment results for diseases associated with aging should be based on biological age rather than CA. Accordingly, ideal biological age indicators should be easily accessible, safe, reliable, and accurately predict morbidity and mortality.

### 4.3. AI-ECG heart age for predicting CVD outcomes

The 12-lead ECG, which is a rapid, simple, reproducible, and inexpensive point-of-care test, is the most used examination for screening and evaluating CVDs (10, 11). ECG is the oldest enduring examination of the heart, excluding the stethoscope, which records the heart's electrical activity of voltage vs. time (25, 26). This examination detects electrical changes resulting from the depolarization and repolarization of the myocardium (27). ECG pattern changes have been described in aging and various cardiac disorders, including cardiac rhythm irregularities, myocardial ischemia and infarctions, ventricular hypertrophy, heart failure, and electrolyte complications (27, 28).

The cardiovascular system may undergo structural and functional changes as the myocardium increases in stiffness because of fibrosis and hypertrophy through aging (29). ECG reveals these structural abnormalities, which are frequently accompanied by structural changes in extracardiac tissues, such as the chest shape, emphysema, and/or increased adiposity (29).

There are frequent changes in the aging electrocardiogram. An increased QRS amplitude, leftward QRS axis, QS pattern due to anatomical changes in heart position, fascicle fibrosis, and senile intraseptal fibrosis, regardless of coronary artery disease or ventricular structural remodeling, are examples of chamber modifications (30). Aging frequently results in repolarization alterations, which are the flattening and depression of the ST segment in the left lateral precordial leads (31). Excluding QT prolongation due to medications, such as antipsychotics, amiodarone, tricyclic antidepressants, antihistamines, and antibiotics, QT prolongation may be increased, but it remains within the normal range in healthy individuals (29). Furthermore, since these medications are frequently used in older patients, they can be considered. Fibrocalcific alterations in the conduction system cause delays and blockades (32).

Some studies used complicated multiple linear regression models that used features of the aging ECG parameters to determine the “ECG heart age.” (33, 34) A recent study has used supervised machine learning and Bayesian statistical approaches to determine the ECG heart age (34). However, these approaches, which use visual and expert analysis measurements, such as the heart rate, R-to-R, P-wave, PR, QRS, QT, and QTc interval durations, as well as the conventional ECG amplitudes and axes, have some components that make it challenging for healthcare personnel to apply these models. Although ECG interpretation requires expert knowledge and experience, the AI-ECG using deep neural networks may discover signals and patterns that are incomprehensible to humans, making it a robust, non-invasive biomarker. Deep learning-based ECG analysis provides several advantages in this respect. Large-scale ECG data are an ideal medical tool for deep neural networks.

Recent studies have indicated that ECG analyses utilizing deep learning approaches to AI-based ECG may predict mortality, cardiac arrhythmias, cardiac function, heart failure, valvular heart disease, and electrolyte abnormalities (7–12, 35, 36). By comparing the ECG age calculated *via* deep neural networks to the CA, new information on mortality and CVD risk factors has recently been derived (8, 13, 36). However, since there is no true gold standard for heart age, more studies and data are needed. Our AI-ECG heart age might provide intuitive information for medical examiners and patients to quickly and easily understand heart health.

CVD frequently progresses without symptoms for many years, making an important contribution to mortality, cardiovascular clinical outcomes, and associated pathological processes (27). Many risk factors for CVD could be reduced by lifestyle modifications such as quitting smoking, changing one's diet, and increasing one's physical activity. The format of the heart age presentation could help patients understand and motivate them regarding their CVD risk (35). Patients changing their lifestyles can contribute to patient-centered care that positively affects the outcome of long-term CVD. In addition, this concept can provide patients and their physicians with further tailored cardiac health information in engaged and motivated patients, which would contribute to the earlier implementation of a better healthy lifestyle.

### 4.4. Predictive value beyond traditional risk factors

Our study revealed that not only traditional risk factors such as chronological age, gender, DM, HTN, HF, stroke, MI, and CKD, but also AI-ECG heart age, were significantly associated with all-cause and cardiovascular-related outcomes. These findings indicate that including AI-ECG heart age may enhance the prognostic value of conventional risk factors for mortality and cardiovascular outcomes. Therefore, AI-ECG heart age may have the potential to serve as an additional and valuable predictor of adverse cardiovascular events beyond the traditional risk factors alone. After adjusting for the relevant risk factors, our study demonstrated that the AI-ECG heart age is associated with all-cause mortality, cardiovascular mortality, and MACE. Lima et al. (8) reported that patients with an ECG age of >8 years greater than the CA have a 1.9-fold higher mortality rate. Attia et al. (14) demonstrated that left ventricular dysfunction and CVD-related comorbidities such as hypertension and coronary artery disease have a significantly higher proportion in patients with an AI-ECG predicted age of >7 years of the CA. Another study reported that patients with an age gap ≥1 standard deviation had higher all-cause mortality and CVD mortality than those whose ECG-derived age was within 1 standard deviation of their CA (13). The MAE of the CA and ECG age in the study by Chang et al. (36) was 6.899 years, and participants with an ECG age of >7 years compared with the CA had a 3.16-fold risk (95% confidence interval: 1.72–5.78) and 1.59-fold risk (95% confidence interval: 1.45–1.74) for all-cause mortality in two different cohorts. In our study, the MAE of the difference between the CA and AI-estimated biological ECG age was 5.8 ± 3.9 years with an *R*-squared of 0.7 (*r* = 0.84, *p* < 0.05). However, when the ECG age was 6 years younger than the CA, the risks of all-cause mortality [HR: 0.82 (0.75–0.91), *p* < 0.05], cardiovascular hospitalization [HR: 0.77 (0.67–0.88), *p* < 0.05], and MACE [HR: 0.78 (0.68–0.89), *p* < 0.05] decreased, but the risk of cardiovascular-related mortality was not statistically significant [HR: 0.87 (0.61–1.25), *p* = 0.46]. We could not determine the precise process by which the AI-ECG heart age was predicted due to the peculiarities of the deep neural networks. Nonetheless, it was interesting that our study results were consistent and similar to those of other deep learning-based ECG age studies despite the different data sets.

The Centers for Disease Control and Prevention in the United States reported the heart age, which is the calculated age of a person's cardiovascular system based on the traditional risk factors, from the Framingham Heart Study (37). In the survey, participants with a calculated heart age of ≥5–7 years than their CA had a 75% increased risk of myocardial infarction, heart failure, or stroke throughout the survey. Interestingly, the biological heart age, based on the AI-ECG rather than the traditional risk factors, consistently increased the mortality at an age gap of approximately 5–8 years. These findings show the possibility that the AI-ECG heart age based on the ECG is comparable to the Framingham risk score for CVD. However, despite the traditional and robust scoring system, Framingham risk scoring for predicting the risk of future cardiac events at the population level does not consider direct physiological information using the questionnaire with indirect tests. Predicted heart age shows regional differences by income level according to a recent study that assessed predicted heart age using individual-level data from 41 World Health Organization STEPS surveys across multiple countries (38).

### 4.5. Future research directions for AI-ECG heart age

Our study demonstrated that as the AI-ECG heart age increased, there were significant alterations in PR interval, QRS duration, QT intervals, and QTc. This suggests that as individuals biological age, there may be changes in their cardiac conduction system and ventricular depolarization and repolarization, which are reflected in ECG changes (39–41). Incorporating AI ECG heart age as a new biomarker could potentially improve risk assessment and guide more effective preventive interventions in patients. It is important to note that although these ECG changes may be age-related, they may also be influenced by other factors such as medication use, underlying medical conditions, and lifestyle factors. Further studies are needed to confirm the association between ECG features and AI-ECG heart age and to evaluate the clinical implications of these findings for predicting cardiovascular outcomes. Additionally, it would be valuable to investigate whether the changes in ECG features associated with AI-ECG heart age could be used to identify individuals at higher risk for cardiovascular disease and to develop interventions to mitigate this risk.

Our study found a non-linear J-shaped association between the gap in the AI-ECG heart age and CA variables and all-cause mortality and cardiovascular risk. Although the exact mechanism is unknown, it has been shown in various medical phenomena (42, 43). Interestingly, the overall J- and linear patterns of the association appear to vary slightly linearly with sex. A higher risk of arrhythmias and diastolic dysfunction in women suggests a relationship between postmenopausal estrogen deficiency and sex hormones (44). Moreover, an animal study suggests that estrogen deficiency may affect diastolic dysfunction through various pathways, including enhanced cardiac remodeling, left ventricular hypertrophy, and increased arteriosclerosis (45). This study's findings suggest the role of sex hormones, including estrogen deficiency, in the etiology of male and female ECG differences. These sex differences appear to result from different linear patterns of cardiovascular outcomes in the AI-ECG age. Furthermore, it has been reported that applying AI to ECG allows for robust detection of the patient's sex (36). However, further studies are needed to better understand the AI-ECG age according to the nature of sex differences.

### 4.6. Limitations

First, this retrospective study was conducted at a tertiary university hospital that comprised mostly healthy Koreans. Although different races and ethnicities were excluded, our results were similar to those of previous AI-ECG age studies, suggesting that the AI-ECG might function regardless of ethnicity. Therefore, larger studies that include diverse races and ethnicities are warranted. Second, considering the inherent constraints of deep neural networks, it was difficult to reach an established definitive “cause-and-effect” conclusion, and there were also technical issues. We conducted training, validation, and holdout testing using >500,000 refined digitalized ECG raw data to minimize these hurdles. Third, the older people in the dataset may not have been a representative sample of the overall population. Therefore, our study may have overlooked the sick and fragile older people. Fourth, since no true gold standard exists for the biological heart age, we could not compare our AI-ECG-based heart age predictions to those of the traditional heart age scoring system or other AI-ECG age studies. Consequently, further research with an acceptable gold standard for heart age is required. Fifth, diagnostic codes were used by the claims database to define the diagnoses due to the nature of the hospital claims data. Several comorbidities and cardiovascular outcomes were based on the International Classification of Diseases codes, which may not always be accurate. Therefore, we plan to integrate ECG data into the nationwide health insurance database to address these issues in the future.

## 5. Conclusions

We developed an AI-ECG age algorithm using large-scale 12-lead ECG data to determine biological age. The difference between the AI-ECG age and CA showed a predictive capability for all-cause mortality, cardiovascular-related mortality, cardiovascular hospitalization, and MACE. Therefore, our model could be useful in understanding and motivating patients regarding their CVD risk, potentially leading to better lifestyle modification to improve the primary prevention of CVD. However, further studies are needed to examine the usefulness of the AI-ECG heart age model in clinical practice.

## Data Availability

The original contributions presented in the study are included in the article/**Supplementary Material**, further inquiries can be directed to the corresponding author/s.
