# Supplementary material for: Artificial intelligence-estimated biological heart age using a 12-lead electrocardiogram predicts mortality and cardiovascular outcomes
Source: Front Cardiovasc Med. 2023 Apr 13;10:1137892. doi: 10.3389/fcvm.2023.1137892 (PMC10133724; doi:10.3389/fcvm.2023.1137892)
Supplement: Supplementary file 1 [file Datasheet1.docx]

Supplementary Material

Artificial intelligence-estimated biological heart age using a 12-lead electrocardiogram predicts mortality and cardiovascular outcomes

Yong-Soo Baek, PhD^†^, Dong-Ho Lee, MS^†^, Yoonsu Jo, MS, Sang-Chul Lee, PhD^*^, Wonik Choi, PhD, Dae-Hyeok Kim, MD^*^

^†^These authors contributed equally to this work and share first authorship.

*** Correspondence:** Sang-Chul Lee: [sclee@inha.ac.kr](mailto:sclee@inha.ac.kr); Dae-Hyeok Kim: [kdhmd@inha.ac.kr](mailto:kdhmd@inha.ac.kr)

# Supplementary Figures


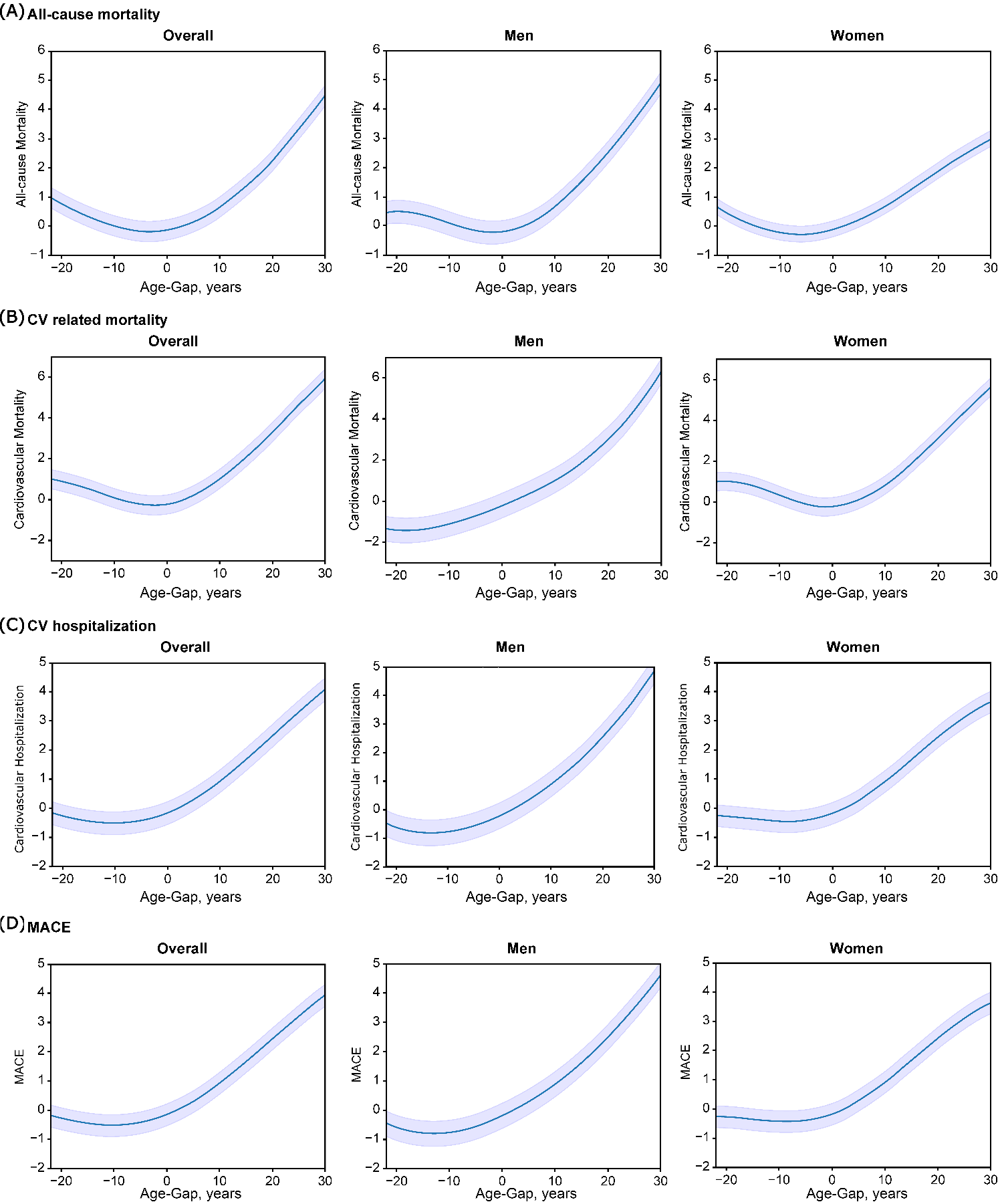


**Supplementary Figure 1.** The relationship between the difference between the AI-estimated ECG age and CA and the mortality and cardiovascular outcomes, using penalized B-spline curves fitted to the Cox proportional hazards model in the overall population and sex-stratified population. AI, artificial intelligence; CA, chronological age; CV, cardiovascular; ECG, electrocardiography; MACE, major adverse cardiovascular events

# Supplementary Figure 2. Correlation Between AI-ECG Heart Age and ECG Findings (A). PR interval, (B). QRS duration, (C) QT interval and (D) QTc
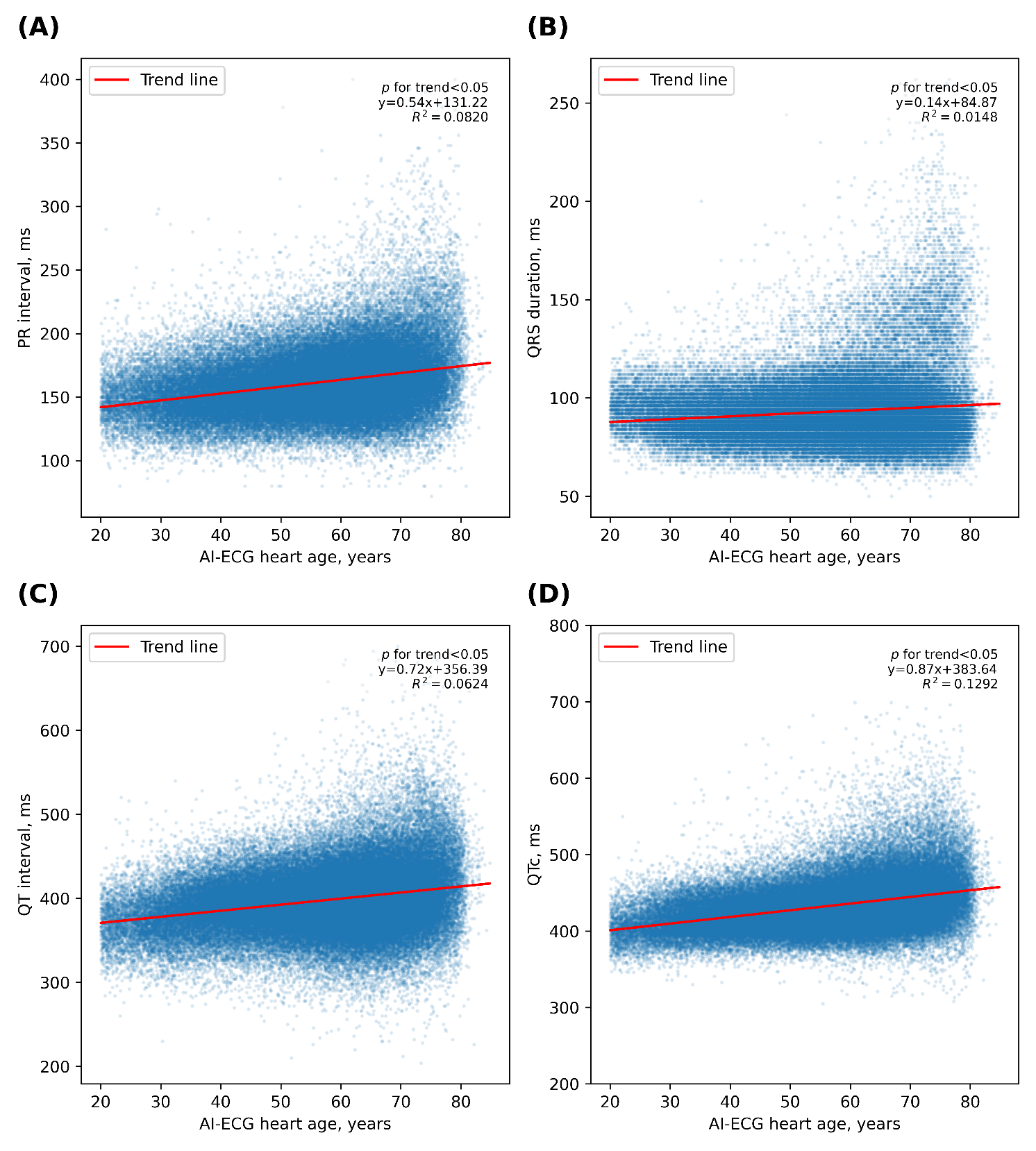


Linear least-squares regression analyses were performed to assess the relationships between AI-ECG heart age and ECG findings including PR interval, QRS duration, QT interval and QTc. The trend line is expressed as y = mx + b (m: slope, b: y-intercept). *p* for trend is made through Wald Test with t-distribution. *R*^2^ means Pearson correlation. AI, artificial intelligence; ECG, electrocardiography; QTc, the corrected QT Interval

# Supplementary Table

# Supplementary Table 1. Univariate hazard ratios for clinical outcomes

|  | **All-cause mortality** | | **Cardiovascular-related mortality** | | **Cardiovascular hospitalization** | | **MACE** | |
| --- | --- | --- | --- | --- | --- | --- | --- | --- |
|  | HR (95% CI) | p-value | HR (95% CI) | p-value | HR (95% CI) | p-value | HR (95% CI) | p-value |
| Chronological age, 1 year | 1.05 (1.04-1.05) | <0.05 | 1.06 (1.05-1.08) | <0.05 | 1.03 (1.03-1.04) | <0.05 | 1.03 (1.02-1.04) | <0.05 |
| AI-ECG heart age, 1 year | 1.05 (1.04-1.05) | <0.05 | 1.08 (1.06-1.10) | <0.05 | 1.04 (1.03-1.05) | <0.05 | 1.04 (1.03-1.05) | <0.05 |
| Chronological age, 5 years | 1.28(1.25-1.31) | <0.05 | 1.37 (1.25-1.49) | <0.05 | 1.18 (1.14-1.22) | <0.05 | 1.17 (1.13-1.21) | <0.05 |
| AI-ECG heart age, 5 years | 1.28 (1.24-1.31) | <0.05 | 1.45 (1.31-1.62) | <0.05 | 1.21 (1.16-1.27) | <0.05 | 1.20 (1.15-1.25) | <0.05 |
| Sex, male | 1.41 (1.26-1.58) | <0.05 | 1.39(0.93-2.07) | 0.11 | 1.34 (1.11-1.61) | <0.05 | 1.32 (1.10-1.59) | <0.05 |
| DM | 1.78 (1.44-2.19) | <0.05 | 5.47 (2.49-12.01) | <0.05 | 1.94 (1.51-2.48) | <0.05 | 1.93 (1.50-2.46) | <0.05 |
| HTN | 1.04 (0.84-1.27) | 0.74 | 1.98(0.93-4.22) | 0.08 | 2.07 (1.62-2.64) | <0.05 | 2.07 (1.62-2.64) | <0.05 |
| HF | 1.90 (1.46-2.46) | <0.05 | 5.57 (2.65-11.72) | <0.05 | 4.44 (3.36-5.86) | <0.05 | 4.39 (3.32-5.79) | <0.05 |
| Stroke (ischemic,  hemorrhagic)/TIA | 1.67 (1.34-2.06) | <0.05 | 1.61 (0.75-3.47) | 0.22 | 1.41 (1.08-1.83) | 0.01 | 1.40 (1.08-1.82) | 0.01 |
| MI | 1.29 (0.92-1.81) | 0.14 | 4.35 (1.92-9.85) | <0.05 | 8.70 (6.27-12.07) | <0.05 | 8.78 (6.33-12.18) | <0.05 |
| CKD | 2.53 (2.00-3.19) | <0.05 | 6.49 (3.12-13.49) | <0.05 | 2.02 (1.48-2.77) | <0.05 | 2.01 (1.47-2.47) | <0.05 |

The hazard ratios (HR) summarize the univariate Cox regression models for all-cause mortality, cardiovascular-related mortality, cardiovascular hospitalization, and MACE. AI, artificial intelligence; CI, confidence interval; CKD, chronic kidney disease; DM, diabetes mellitus; ECG, electrocardiography; HF, heart failure; HTN, hypertension; HR, hazard ratio; MACE, major adverse cardiovascular events; MI, myocardial infarction; NA, not applicable; TIA, transient ischemic attack

**Supplementary Table 2.** Analysis of ECG features based on AI-ECG heart age

| AI-ECG heart age | < 29 years | 30-39 years | 40-49 years | 50-59 years | 60-69 years | ≥70 years | p-value |
| --- | --- | --- | --- | --- | --- | --- | --- |
| PR interval, ms | 148.06 ± 19.69 | 150.68 ± 20.26 | 155.38 ± 21.53 | 159.83 ± 23.44 | 163.67 ± 27.24 | 173.54 ± 36.97 | <0.05 |
| QRS duration, ms | 92.55 ± 10.55 | 90.01 ± 10.67 | 90.48 ± 11.48 | 91.74 ± 12.91 | 93.20 ± 16.33 | 97.82 ± 24.79 | <0.05 |
| QT interval, ms | 372.31 ± 28.32 | 383.70 ± 30.76 | 392.02 ± 32.61 | 395.15 ± 35.90 | 400.33 ± 41.83 | 413.26 ± 49.18 | <0.05 |
| QTc, ms | 409.05 ± 23.13 | 417.93 ± 23.85 | 422.79 ± 25.29 | 429.26 ± 28.99 | 439.04 ± 34.43 | 451.47 ± 40.62 | <0.05 |

Values are expressed as means ± standard deviations. ANOVA test was used to determine statistical significance among AI-ECG heart age groups. AI, artificial intelligence; ECG, electrocardiography; QTc, the corrected QT Interval
